# Genetic Variants of the *PLCXD3* Gene Are Associated with Risk of Metabolic Syndrome in the Emirati Population

**DOI:** 10.3390/genes11060665

**Published:** 2020-06-18

**Authors:** Hayat Aljaibeji, Abdul Khader Mohammed, Sami Alkayyali, Mahmood Yaseen Hachim, Hind Hasswan, Waseem El-Huneidi, Jalal Taneera, Nabil Sulaiman

**Affiliations:** 1 Sharjah Institute for Medical Research, University of Sharjah, Sharjah 27272, UAE; hayat_aljaibeji@yahoo.com (H.A.); amohammed@sharjah.ac.ae (A.K.M.); hind.smsm@gmail.com (H.H.); 2 Laboratory of Clinical Chemistry and Transfusion Medicine, Central Hospital of Växjö, Växjö 35188, Sweden; samikayyali@hotmail.com; 3 College of Medicine, Mohammed Bin Rashid University of Medicine and Health Sciences, Dubai 505055, UAE; drmahmoodhachim@gmail.com; 4 Department of Basic Medical Sciences, College of Medicine, University of Sharjah, Sharjah 27272, UAE; welhuneidi@sharjah.ac.ae; 5 Department of Family Medicine, College of Medicine, University of Sharjah, Sharjah 27272, UAE; 6 Baker IDI Heart and Diabetes Institute, Melbourne 3004, Australia

**Keywords:** phosphatidylinositol-specific phospholipase C X domain, HbA1c, type 2 diabetes, MetS, metabolic syndrome, MAF, minor allele frequency, BMI, body mass index, LDL, triglycerides, CJD, single-nucleotide polymorphism, SBP, diastolic blood pressure

## Abstract

Phosphatidylinositol-specific phospholipase C X domain 3 *(PLCXD3)* has been shown to influence pancreatic β-cell function by disrupting insulin signaling. Herein, we investigated two genetic variants in the *PLCXD3* gene in relation to type 2 diabetes (T2D) or metabolic syndrome (MetS) in the Emirati population. In total, 556 adult Emirati individuals (306 T2D and 256 controls) were genotyped for two *PLCXD3* variants (rs319013 and rs9292806) using TaqMan genotyping assays. The frequency distribution of minor homozygous CC genotype of rs9292806 and GG genotype of rs319013 were significantly higher in subjects with MetS compared to Non-MetS (*p* < 0.01). The minor homozygous rs9292806-CC and rs319013-GG genotypes were significantly associated with increased risk of MetS (adj. OR 2.92; 95% CI 1.61–5.3; *p* < 0.001) (adj. OR 2.62; 95% CI 1.42–4.83; *p* = 0.002), respectively. However, no associations were detected with T2D. In healthy participants, the homozygous minor genotypes of both rs9292806 and rs319013 were significantly higher fasting glucose (adj. *p* < 0.005), HbA1c (adj. *p* < 0.005) and lower HDL-cholesterol (adj. *p* < 0.05) levels. Data from T2D Knowledge Portal database disclosed a nominal association of rs319013 and rs9292806 with T2D and components of MetS. Bioinformatics prediction analysis showed a deleterious effect of rs9292806 on the regulatory regions of *PLCXD3*. In conclusion, this study identifies rs319013 and rs9292806 variants of *PLCXD3* as additional risk factors for MetS in the Emirati population.

## 1. Introduction

Metabolic syndrome (MetS) is a major health problem, referring to cluster risk factors that include obesity, dyslipidemia, hyperglycemia and hypertension [1,2,3]. Components of MetS, individually or collectively, increase the risk of type 2 diabetes mellitus (T2D) and cardiovascular (CVD) diseases [4,5,6]. The International Diabetes Federation (IDF) estimates that a quarter of the adult population worldwide suffers from MetS [1]. The national estimates of MetS among adults in the United Arab Emirates (UAE) have reached to 40 percent [7], with nearly 75 percent of the population either overweight or obese [8]. Aside from lifestyle factors and physical inactivity, genetics is considered as an essential risk factor for metabolic syndrome [9]. 

Recently, we showed that the expression of *PLCXD3*, a member of the PI-PLC family, is downregulated in human diabetic islets, inversely correlated with HbA1c and positively correlated with insulin secretion [10,11]. Further investigations revealed that *PLCXD3* is involved in insulin signaling and glucose sensing, suggesting that *PLCXD3* might be regarded as a candidate gene for pre-diabetes and metabolic syndrome. Despite the role of *PLCXD3* in β-cell function, until now no studies have linked genetic variants in the *PLCXD3* gene with T2D, MetS or its related traits. 

Thus, the present study aims to investigate the association of two intronic SNPs “rs319013 and rs9292806” with T2D or MetS in the Emirati population.

## 2. Materials 

### 2.1. Study Population

In total, 556 unrelated adult Emirati participants (306 T2D (120 males and 186 females)) and 256 controls (119 males and 137 females) were selected from two different cohorts were included for this study. The first cohort consisted of participants from UAE national diabetes and lifestyle study (UAEDIAB) that includes participants living in Dubai, Sharjah, and the Northern Emirates collected from door to door visits as described previously [12,13,14]. The second cohort includes participants from the All-New Diabetes in Sharjah and Ajman (ANDISA) study were patients recruited to this study based on their routine visit to the endocrinology clinic at the university hospital of Sharjah. The initial study was approved by the UAE ministry of health (MOHAP/DXB/SUBC/No.14/2017) and University of Sharjah ethics committee. A written informed consent with an extensive interview and a standard questionnaire were obtained from all the participants. Using the International Diabetes Federation (IDF) criteria for metabolic syndrome [3], the studied participants were re-classified into MetS and Non-MetS. The features for MetS include a waist circumference ≥ 102 cm for men and ≥ 88 cm for women, blood pressure ≥ 130/85, fasting plasma glucose levels ≥ 5.6 mmol/L, HDL-cholesterol < 40 mg/dL for men and < 50 mg/dL for women and triglycerides ≥ 1.7 mmol/L. The MetS is defined as central obesity plus other two factors. For participants without waist circumference data, BMI ≥30 kg/m^2^ were assumed as central obesity. Subjects who did not match the employed criteria for MetS selection were considered as Non-MetS. 

All participants were requested to provide information on demographics, medical and family history of diabetes and current medications. Anthropometric parameters, including height, weight, mean systolic blood pressure, and diastolic blood pressure (average of three readings) were obtained from all the participants. Body mass index (BMI) values were computed by dividing weight in kilograms by height in meter square. Fasting blood samples were collected from participating individuals for a glucose test, HbA1c, and lipid profile. The same blood samples were used later for DNA extraction. 

### 2.2. Genotyping Analysis

**Figure 1:**
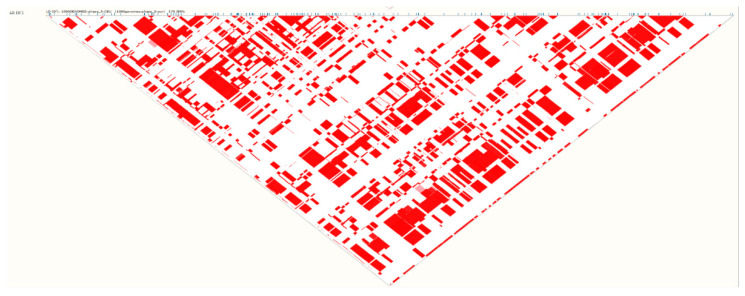
Linkage disequilibrium rs319013 and rs9292806 (D′ = 1.0).

**Figure 2:**
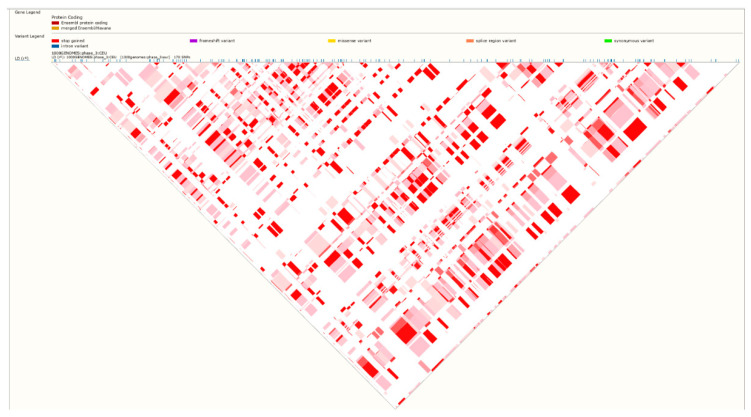
Linkage disequilibrium rs319013 and rs9292806 (*r*^2^ = 0.977).

The genomic DNA was extracted from whole blood using pure link genomic DNA mini Kit (Invitrogen, Carlsbad, CA, USA). DNA concentration and purity were checked by Nano-drop 2000 C spectrophotometer (Thermo Scientific, Wilmington, NC, USA). Two tagging SNPs (Intronic variants) in *PLCXD3* gene rs319013 and rs9292806 have been selected for genotyping. Both rs319013 and rs9292806 were in very high linkage disequilibrium, for example, in the north European population [15] *r*^2^ = 0.977 and D′ = 1.0 (Figure 1 and Figure 2). Data from 1000 Genomes Phase 3 showed that the combined population minor allele frequency of rs319013 is 0.43 and 0.44 for rs9292806. The distance between the SNPs is about 34-kilo base pairs (kbp) (https://jan2020.archive.ensembl.org/Homo_sapiens/Location/LD?db=core;focus=variation;pop1=373514;r=5:41382400-41416900;v=rs319013;vdb=variation;vf=49567201). The genotyping was performed by allelic discrimination real-time PCR using TaqMan assays for genotyping (Applied Biosystems, Foster City, CA, USA). The assay IDs are C_805815_10 for rs319013 and C__30418796_20 for rs9292806. All qPCR amplifications were carried out in a final reaction volume of 10 µL containing 1X firepol universal probe master mix (Solis Biodyne, Tartu, Estonia), 1X TaqMan genotyping assays, and 50 ng of template DNA. All amplifications and detections were conducted on genomic DNA in 96-well PCR plates using a QuantStudio three Real-time PCR (Applied Biosystems, Foster City, CA, USA). A minimum of two non-template control was included in each run. Thermal cycling was initiated with pre-PCR read followed by a denaturation step of 10 min at 95 °C followed by 50 cycles of 15 s at 95 °C, 60 s at 60 °C. Allelic discrimination analysis was performed using QuantStudio Real-Time PCR Software autocaller (Thermo Fisher, Waltham, MA, USA).

### 2.3. Statistical Analyses

All the statistical analyses were carried out with SPSS version 26 (IBM, Armonk, NY, USA). The Hardy–Weinberg equilibrium was tested using a Chi-square test. Linkage disequilibrium was calculated using haploview. Haplotype frequencies were estimated by an Expectation–Maximization algorithm (EM algorithm) with haploview software [16]. The most common haplotype was used as the reference. The non-Gaussian variables are presented as median with interquartile range. An independent sample *t*-test was used to compare the difference between the groups, while the Mann–Whitney U test was used for comparison of nonparametric variables. The genotype frequency differences between the categorized group (control vs. T2D or Non-MetS vs. MetS) were tested using a chi-square test. Odds ratios (ORs) with 95% confidence intervals (CIs) were estimated by multinomial logistic regression with age and gender as covariates. The major allele was employed as the reference genotype. Analysis of variance (ANOVA) was used to compare different genotypic groups with anthropometric and biochemical parameters followed by application of Bonferroni post hoc test, while univariate general linear model (GLM) was used for adjusting covariates such as age and gender. The significance was set at *p*-value <0.05. All the continuous normal variables are presented as mean ± standard deviation (SD).

## 3. Results

The anthropometric and clinical variables of the studied participants for control vs. T2D groups were shown in Table 1 and Non-MetS vs. Mets are presented in Table 2. Measurements of BMI, waist circumference SBP, HbA1c, fasting glucose and triglycerides were significantly higher in T2D and MetS groups when compared to controls (*p* < 0.001), while levels of lipids profile (total cholesterol, LDL- and HDL-cholesterol) were, in general, lower. 

**Figure 3:**
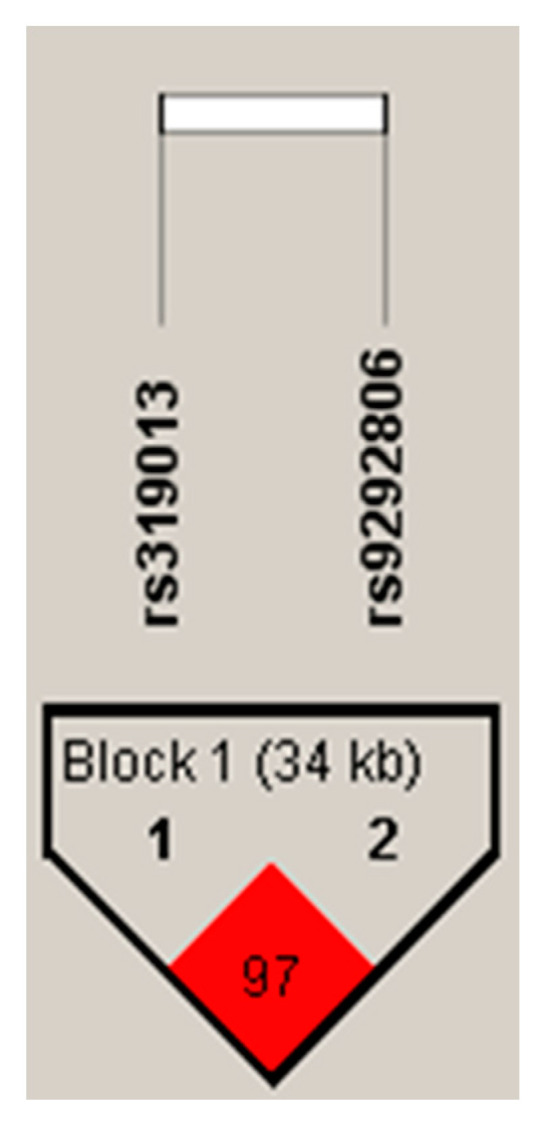
Linkage disequilibrium (*r*^2^ = 0.972) analysis between rs319013 and rs9292806 in the Emirati population, *r*^2^ indicates the squared correlation coefficient between two SNPs.

The genotype frequency distribution of both rs319013 and rs9292806 in the control and Non-MetS group were consistent with the Hardy–Weinberg equilibrium (*p* > 0.05). The genotype frequency distribution of both rs319013 and rs9292806 between T2D and control study groups are described in Table 3. No significant difference in genotype frequencies was found between control and T2D group. However, the genotype frequencies of rs319013 and rs9292806 were significantly different between MetS and Non-MetS subjects (Table 3, *p* < 0.05). The frequency of homozygous CC genotype of rs9292806 was significantly higher in individuals with MetS than Non-MetS (MetS 18% vs. Non-MetS 8%) (Table 3). Similarly, the frequency of homozygous GG genotype of rs319013 was significantly higher in individuals with MetS than Non-MetS (Mets 16% vs. controls 8.1%). The association of the *PLCXD3* gene variants towards a predisposition to T2D or MetS was analyzed by multiple logistic regression considering age and gender as potential covariates. Our results indicated that both of the studied SNPs were not associated with risk of T2D (Table 4). However, the homozygous CC genotype of rs9292806 and homozygous GG genotype of rs319013 were significantly associated with risk of MetS (adj. OR 2.92; 95% CI 1.61–5.3; *p* < 0.001) (adj. OR 2.62; 95% CI 1.42–4.83; *p* = 0.002), respectively (Table 4). Furthermore, we investigated the association of the two SNPs rs319013 and rs9292806 with anthropometric parameters in control subjects. As shown in Table 5, the homozygous genotypes of both rs319013 (GG) and rs9292806 (CC) showed statistically significant higher levels of fasting glucose levels (adj. *p* < 0.05), HbA1c (adj. *p* < 0.01) and lower HDL-cholesterol (adj. *p* <0.01) levels (Table 5). Linkage disequilibrium analysis of our studied population indicated both rs319013 and rs9292806 were in very high linkage disequilibrium (*r*^2^ = 0.972) (Figure 3). The frequency distribution of rs319013_G and rs9292806_C (i.e., GC) haplotype is more common MetS group compared to Non-MetS group, thus GC haplotype is associated with increased risk of MetS (OR 1.46; 95% CI 1.01–2.14); *p* = 0.047) (Table 6). However, no such differences were found in control vs. T2DM group (Table 6).

### 3.1. Association of rs319013 and rs9292806 across GWAS Datasets with T2D and Related Traits

The T2D Knowledge Portal (T2DKP; contains 88 datasets and 198 traits) database (www.type2diabetesgenetics.org) was used to explore GWAS datasets for the association of rs319013 and rs9292806 with T2D and other traits. As shown in Table 7, we detected nominally significant associations (*p* < 0.05) between the variant allele of *PLCXD3* rs319013 with BMI, creatinine, diastolic blood pressure, eGFR-creat (serum creatinine), HbA1c, height, LDL cholesterol, pericardial adipose tissue volume, triglycerides and T2D in several datasets. However, the most significant associations were observed with BMI (*p* < 0.00066) in BioBank Japan GWAS, males dataset and T2D (*p* < 0.00064) in AMP T2D-GENES T2D exome sequence analysis dataset. The variant allele rs9292806 was nominally associated (*p* < 0.05) with adiponectin, BMI, eGFR-creat, height, pericardial adipose tissue volume, triglycerides, T2D (Table 8). Likewise, the most significant associations of rs9292806 were observed with BMI (*p* < 0.0008) in BioBank Japan GWAS, male dataset and height (*p* < 0.006) in GIANT UK Biobank GWAS dataset. These data provide more evidence for the association of rs319013 and rs9292806 in MetS disorders.

### 3.2. Prediction the Effect of rs319013 and rs9292806 on the Function of PLCXD3

**Figure 4:**
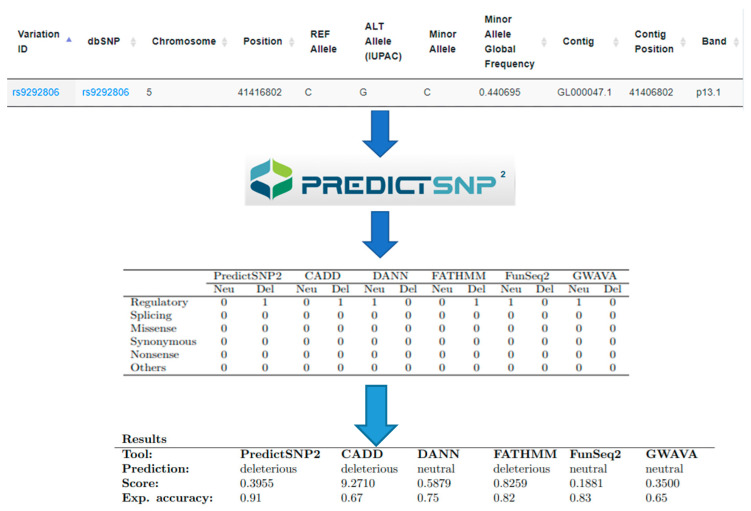
Prediction the effect of the rs9292806 of function and expression of *PLCXD3* using the PredictSNP2 platform.

To predict the possible consequences of the examined SNPs on the function or expression of the *PLCXD3*, the chromosomal location for two *PLCXD3* variants (rs319013 and rs9292806), reference allele and altered allele were used in online tools “PredictSNP2” (https://loschmidt.chemi.muni.cz/predictsnp2/) [25]. PredictSNP2 is a unified platform for accurately evaluating SNP effects by exploiting the different characteristics of variants in distinct genomic regions. As shown in Figure 4, only rs9292806 showed a deleterious effect on regulatory regions using PriedictSNP2, CADD and FATHMM prediction tools with an expected accuracy of 91%, 67 % and 82 %, respectively.

## 4. Discussion and Conclusions

It is well established that MetS increase the risk for cardiovascular disease, T2D and other conditions include dyslipidemia, high blood pressure, excess body fat around the waist and high fasting plasma glucose [26,27,28]. MetS is ascribed to an interaction between genetic and environmental factors like obesity and lifestyle [29,30,31]. As the prevalence of MetS disease is expected to escalate globally, identification of genetic markers could be an early prediction to minimize the risk of MetS, T2D and cardiovascular diseases.

In this study, we examined the association of genetic variants of the *PLCXD3* gene (rs319013 and rs9292806) with T2D or MetS among UAE nationals. Our results revealed the presence of an association between the homozygous minor genotypes CC-rs9292806 and GG-rs319013 with increased risk of MetS but not T2D (Table 4). GWAS data from T2DKP revealed a significant association of rs9292806 and rs319013 with T2D, BMI and other MetS components in European and Japanese populations (Table 7 and Table 8). The finding that both variants have similar association is not surprising as both displayed a very high linkage disequilibrium (Figure 1 and Figure 2).

The association of *PLCXD3* variants with fasting glucose or HbA1c in our control subjects (Table 5) is supported by a previously published data set [17,22]. While other reports showed no association between *PLCXD3* (rs319013) with T2D [32,33]. Other datasets indicated a statistically significant association of rs319013 with T2D, as shown in Table 7 and Table 8 [19,24,34,35].

To the best of our knowledge, this is the first report investigating the association of genetic variants in the *PLCXD3* gene with T2D or MetS, particularly in the UAE population. In a previous study, genetic variants in the *PLCXD3* were linked with an early onset bipolar disorder vulnerability and olfactory sensory neurons and CJD [36,37,38]. The latter finding was disputed by another report [39]. Moreover, a mutation in the *PLCXD3* gene was associated with rapid-onset obesity with hypothalamic dysfunction, hypoventilation and autonomy dysregulation (ROHHAD) [40]. The latter finding is in line with the association of rs319013 and rs9292806 with BMI (Table 7 and Table 8).

PI-PLC is an enzyme that hydrolyzes the membrane phospholipid phosphatidylinositol-4,5-bisphosphate (PIP_2_) to inositol-1,4,5-trisphosphate (IP_3_) and diacylglycerol in response to external stimuli such as hormones, neurotransmitters and growth factors [41]. Each PI-PLC subtype contains a well-conserved catalytic domain of separate X- and Y-box. In contrast, the *PLXCD* isoforms (*PLCXD1, PLCXD2* and *PLCXD3*) have only the catalytic X domain with distinct functions, various tissue distribution and cellular localization [42]. 

*PLCXD3* is highly expressed in human pancreatic islets [10], significantly downregulated in diabetic islets, correlated positively with insulin secretion and negatively with HbA1c as well as BMI [10]. This is in line with our data showing the homozygous genotype of rs9292806 (CC) and rs319013 (GG) have a significantly higher glycemic profile represented by fasting blood glucose and HbA1c in control subjects. 

The mechanisms by which these genetic variants affect glucose hemeostasis is not clear. However, it can be speculated that these variants influence the expression of *PLCXD3*, in turn, *PLCXD3* affects the glycemic profile. Despite that fact that rs319013 is intronic, it lies at the junction of intron 1 and exon 2 in close proximity to the splice site motifs [36,39]. As exon 2 codes for the active structural domain of *PLCXD3* protein, hence any modification to the functioning of the spliceosome at this particular region might impact the activity of the *PLCXD3* protein [36,39] and might be influencing the expression of *PLCXD3* by altering the mRNA stability or binding of transcription factors. In line with this hypothesis, we showed a bioinformatics tool that rs9292806 influences the regulatory regions of *PLCXD3*. A possible validation for this finding is to investigate the mRNA expression of *PLCXD3* among our participants’ samples with different genotypes. Unfortunately, due to the shortage of RNA materials, we could not perform such analysis. 

We believe that it is crucial to replicate the association of the studied variants as well as other variants within the *PLCXD3* gene in different ethnic populations. More, the expression level of *PLCXD3* needs to be explored in various tissues among different pathological conditions related to metabolic syndrome such as fat, heart, muscle, and brain tissue. 

In conclusion, rs9292806 and rs319013 in the *PLCXD3* gene are associated with MetS but not T2D in the Emirati population. The finding emphasizes the power of genetic susceptibility to use as biomarkers for prevention strategy of MetS in UAE. Further studies with larger sample sizes and subgroups are warranted for validation and replication. 

## Figures and Tables

**Table 1:** Anthropometric and clinical characteristics of the studied groups.

Parameters	Control (*n* = 256)	T2DM (*n* = 306)	*p* Value
N (M/F)	119/137	120/186	
Age (Years)	43.3 ± 12.7	54.4 ± 10.8	<0.0001
BMI (kg/m^2^)	28.8 ± 5.2	31.2 ± 5.8	<0.0001
Waist circumference	96.4 ± 12.9	103.5± 12.6	0.001
SBP (mmHg)	125.3 ± 17.9	132.1 ± 16.2	<0.0001
DBP (mmHg)	77.6 ± 10.7	77.7 ± 10.3	0.89
Glucose (mmol/L)	5.35 ± 0.66	10.0 ± 3.48	<0.0001
HbA1c (%)	5.44 ± 0.48	8.47 ± 1.51	<0.0001
Total Cholesterol (mmol/L)	5.0 ± 0.94	4.6 ± 1.36	<0.0001
HDL-Cholesterol (mmol/L)	1.39 ± 0.45	1.24 ± 0.36	<0.0001
LDL-Cholesterol (mmol/L)	3.20± 0.82	2.78 ± 1.06	<0.0001
Triglycerides (mmol/L) ^#^	1.1 (0.81–1.57)	1.36 (1.05–1.92)	<0.0001

Data were presented as mean ± standard deviation for normal continuous variables; ^#^ denotes continuous variables with non-Gaussian distribution and presented as median (1st–3rd quartile). Independent sample *t*-test and a Mann–Whitney U test were used to test differences between control and T2DM groups. Note: Waist circumference data available for 150 participants.

**Table 2:** Anthropometric and clinical characteristics of the studied groups.

Parameters	Non-MetS (*n* = 341)	MetS (*n* = 215)	*p* Value
N (M/F)	161/180	74/141	
Age (Years)	46.5 ± 13.7	53.8 ± 10.1	<0.0001
BMI (kg/m^2^)	26.9 ± 4.0	34.9 ± 4.3	<0.0001
Waist circumference	93.5 ± 11.9	109.2 ± 8.8	<0.0001
SBP (mmHg)	125.2 ± 16.6	132.7 ± 16.7	<0.0001
DBP (mmHg)	76.3 ± 10.7	80.0 ± 9.6	<0.0001
Glucose (mmol/L)	7.0 ± 3.02	8.1 ± 3.55	<0.0001
HbA1c	6.62 ± 1.82	7.79 ± 1.79	<0.0001
Total Cholesterol (mmol/L)	4.84 ± 1.22	4.67 ± 1.17	0.15
HDL-Cholesterol (mmol/L)	1.37 ± 0.44	1.22 ± 0.34	<0.0001
LDL-Cholesterol (mmol/L)	3.01± 1.02	2.90 ± 0.93	0.08
Triglycerides (mmol/L) ^#^	1.12 (0.84–1.68)	1.41 (1.06–2.14)	0.001

Data were presented as mean ± standard deviation for normal continuous variables; ^#^ denotes continuous variables with non-Gaussian distribution and presented as median (1st quartile-3rd quartile). Independent sample *t*-test and a Mann-Whitney U test were used to test differences between control and T2DM groups. Note: Waist circumference data available for 150 participants.

**Table 3:** Genotype frequency distribution of *PLCXD3* SNPs in Control vs. T2DM and Non-MetS vs. MetS subjects.

	Control N (%)	T2DM N (%)	Chi^2^ *p* Value	Non-MetS N (%)	MetS N (%)	Chi^2^ *p* Value
rs9292806						
GG	125 (49.0)	169 (56.1)	0.21	188 (55.5)	104 (49.3)	0.002
CG	99 (38.8)	97 (32.2)		124 (36.6)	69 (32.7)	
CC	31 (12.2)	35 (11.6)		27 (8)	38 (18.0)	
rs319013						
TT	124 (49.2)	171 (57.0)	0.18	187 (56.0)	106 (50.0)	0.015
GT	97 (38.5)	98 (32.7)		120 (35.9)	72 (34.0)	
GG	31 (12.3)	31 (10.3)		27 (8.1)	34 (16.0)	

Genotype frequency differences between Control vs. T2DM and Non-MetS vs. MetS groups were tested using Chi-square test.

**Table 4:** Odds ratios of genotypes the *PLCXD3* SNPs in control vs. T2DM and Non-MetS vs. MetS groups.

	T2D OR (95 % CI)	*p* Value	T2D Adj OR (95 % CI)	Adj *p* Value	MetS OR (95 % CI)	*p* Value	MetS Adj OR (95 % CI)	Adj *p* Value
**rs9292806**								
**GG**	1	-	1	-	1	-	1	-
**CG**	0.72 (0.50–1.10)	0.08	0.67 (0.43–1.03)	0.07	1.01 (0.69–1.47)	0.97	1.03 (0.69–1.55)	0.85
**CC**	0.83 (0.49–1.42)	0.51	0.80 (0.42–1.49)	0.47	2.54 (1.47–4.40)	**0.001**	2.92 (1.61–5.30)	<0.001
**rs319013**								
**TT**	1	-	1	-	1	-	1	-
**GT**	0.73 (0.51–1.05)	0.10	0.66 (0.36–1.02)	0.07	1.06 (0.72–1.54)	0.77	1.08 (0.72–1.63)	0.67
**GG**	0.72 (0.42–1.25)	0.25	0.69 (0.36–1.33)	0.28	2.22 (1.27–3.88)	**0.005**	2.62 (1.42–4.83)	0.002

Odds ratios (ORs) and 95 % confidence intervals for genotypes were calculated using multinomial logistic regression analyses. Adj OR denotes ORs after adjusting for age and gender. The most common genotype was used as the reference genotype. Significant *p* values are bolded.

**Table 5:** Distribution of anthropometric and biochemical parameters according to *PLCXD3* SNPs in control participants (*n* = 256).

	rs9292806					rs319013				
Variables	GG (125)	CG (99)	CC (29)	*p* Value	*p* adj	TT (124)	GT (97)	GG (29)	*p* Value	*p* adj
**Age (Years)**	43.5 ± 13.2	42.6 ± 12.3	44.8 ± 12.2	0.72	-	43.1 ± 13.5	42.8 ± 12.1	44.8 ± 12.3	0.75	-
**BMI (kg/m^2^)**	28.9 ± 5.7	28.4 ± 4.6	29.7 ± 4.7	0.51	0.57	28.9 ± 5.7	28.3 ± 4.6	29.5 ± 4.7	0.44	-
**Waist Circumference**	97.2 ± 12.4	93.1 ± 14.2	102.8 ± 7.3 ^b^	0.03	0.16	97.1 ± 12.4	93.0 ± 14.1	102.7 ± 7.3 ^b^	0.03	0.16
**SBP (mmHg)**	123.0 ± 16.0	126.0 ± 18.2	133.1 ± 21.9 ^a^	0.02	0.20	122.5 ± 17.9	126.2 ± 18.3	133.5 ± 22.2 ^a^	0.01	0.16
**DBP (mmHg)**	76.1 ± 9.8	78.7 ± 11.4	80.3 ± 11.2	0.07	0.19	76.2 ± 9.8	78.6 ± 11.7	79.9 ± 11.2	0.12	0.34
**Glucose (mmol/L)**	5.29 ± 0.58	5.30 ± 0.63	5.75 ± 0.85 ^a,b^	0.006	0.003	5.30 ± 0.58	5.42 ± 0.64	5.72 ± 0.87 ^a,b^	0.013	0.009
**HbA1c (%)**	5.40 ± 0.43	5.42 ± 0.45	5.75 ± 0.67 ^a,b^	0.002	0.004	5.40 ± 0.43	5.41 ± 0.46 ^a^	5.76 ± 0.67 ^a,b^	0.001	0.003
**Total Chol (mmol/L)**	4.91 ± 0.90	5.0 ± 0.94	5.31 ± 1.06	0.16	0.20	4.92 ± 0.90	4.98 ± 0.94	5.31 ± 1.06	0.17	0.22
**HDL-Chol (mmol/L)**	1.50 ± 0.51	1.34 ± 0.35 ^a^	1.12 ± 0.34 ^a,b^	0.001	0.02	1.49 ± 0.51	1.34 ± 0.35 ^a^	1.15 ± 0.37 ^a,b^	0.002	0.03
**LDL-Chol (mmol/L)**	3.12 ± 0.76	3.23 ± 0.85	3.45 ± 0.92	0.17	0.29	3.13 ± 0.76	3.24 ± 0.85	3.42 ± 0.95	0.25	0.37
**Triglycerides (mmol/L) #**	1.29 ± 0.84	1.25 ± 0.70	1.98 ± 1.41 ^a,b^	0.02	0.32	1.30 ± 0.84	1.26 ± 0.71	1.90 ± 1.44	0.10	0.73

Data presented as mean ± standard deviation. # denotes values were log-transformed prior to analysis. *p* adj indicates *p* values after adjusting for age and gender. Superscript ^a^ indicates significantly different from homozygous major genotype group (GG-rs9229806 or TT-rs319013). Superscript ^a,b^ significantly different from homozygous major and heterozygote genotype groups. Note: Waist circumference data available for 97 participants.

**Table 6:** Haplotype frequency of *PLCXD3* variants (rs319013, rs9292806) in Control vs. T2DM and Non-MetS vs. MetS subjects.

Haplotypes.	Haplotype Count		Haplotype Frequencies		OR (95 % CI)	*p* Value
	**Control**	**T2DM**	**Control**	**T2DM**		
TG	175	222	0.68	0.73	1	
GC	81	84	0.32	0.27	0.82 (0.56–1.18)	0.28
	**Non-MetS**	**MetS**	**Non-MetS**	**MetS**		
TG	252	142	0.74	0.66	1	-
GC	89	73	0.26	0.34	1.46 (1.01–2.14)	0.047

**Table 7:** Association of rs319013 across all datasets and traits included in the Type2Diabetes knowledge Portal.

Trait	Dataset	*p*-Value	Direction of Effect	Odds Ratio	MA Frequency	Effect	Samples	References
BMI	BioBank Japan GWAS, males	0.00663	↓			−0.0132	85894	[17]
Creatinine	GoDartsAffymetrix GWAS	0.044	↓		0.379	−0.0546	2917	[18]
Diastolic blood pressure	13K exome sequence analysis	0.0186	↓			−0.0326	12954	[19]
eGFR-creat (serum creatinine)	Hoorn DCS 2018	0.029	↓		0.37	−0.0551	3414	[20]
eGFR-creat (serum creatinine)	SUMMIT Diabetic Kidney Disease GWAS	0.041	↓			−0.82	40340	[21]
HbA1c	MAGIC HbA1c GWAS: Europeans	0.0425					123665	[22]
Height	GIANT UK Biobank GWAS	0.0015	←			0.0047	79564	[23]
LDL cholesterol	BioBank Japan GWAS	0.0455	←			0.0105	191764	[17]
Pericardial adipose tissue volume	VATGen GWAS	0.012	←				18332	[22]
Triglycerides	BioBank Japan GWAS	0.0485	←			0.0085	191764	[17]
Type 2 diabetes	AMP T2D-GENES T2D exome sequence analysis	0.00642	↓	0.954			49147	[19]

**Table 8:** Association of rs9292806 across all datasets and traits included in the Type2Diabetes knowledge Portal.

Trait	Dataset	*p*-Value	Direction of Effect	Odds Ratio	MA Frequency	Effect	Samples	References
Adiponectin	ADIPOGen GWAS	0.0425	←		0.0333	0.00976	45891	[15]
BMI	BioBankJapan GWAS, males	0.00898	↓		0.433	−0.0131	85894	[17]
eGFR-creat (serum creatinine)	Hoorn DCS 2018	0.028	↓		0.361	−0.0573	3414	[20]
eGFR-creat (serum creatinine)	SUMMIT Diabetic Kidney Disease GWAS	0.035	↓		0.38	−0.86	4034	[21]
Height	GIANT UK Biobank GWAS	0.0062	←			0.0041	795640	[23]
Pericardial adipose tissue volume	VATGen GWAS	0.016	←				18332	[22]
Triglycerides	BioBank Japan GWAS	0.0369	←		0.43	0.00962	191764	[17]
Type 2 diabetes	UK Biobank T2D GWAS (DIAMANTE-Europeans 2018)	0.032	↓	0.977	0.4		442817	[24]

## Abbreviations

*PLCXD3*	phosphatidylinositol-specific phospholipase C X domain
HbA1c	glycated hemoglobin
T2D	Type 2 diabetes
MetS	metabolic syndrome
MAF	minor allele frequency
BMI	body mass index
LDL	low-density lipoprotein
HDL	high-density lipoprotein
TG	triglycerides
CJD	sporadic Creutzfeldt-Jakob disease
SNP	single-nucleotide polymorphism
SBP	ystolic blood pressure
DBP	diastolic blood pressure

## References

[1] Alberti K.G., Eckel R.H., Grundy S.M., Zimmet P.Z., Cleeman J.I., Donato K.A., Fruchart J.C., James W.P., Loria C.M., Smith S.C. (2009). Harmonizing the metabolic syndrome: A joint interim statement of the International Diabetes Federation Task Force on Epidemiology and Prevention; National Heart, Lung, and Blood Institute; American Heart Association; World Heart Federation; International Atherosclerosis Society; and International Association for the Study of Obesity. Circulation.

[2] Chang B.C.-C., Hwang L.-C., Huang W.-H. (2018). Positive Association of Metabolic Syndrome with a Single Nucleotide Polymorphism of Syndecan-3 (rs2282440) in the Taiwanese Population. Int. J. Endocrinol..

[3] Alkharfy K.M., Al-Daghri N.M., Al-Attas O.S., Alokail M.S., Mohammed A.K., Vinodson B., Clerici M., Kazmi U., Hussain T., Draz H.M. (2012). Variants of endothelial nitric oxide synthase gene are associated with components of metabolic syndrome in an Arab population. Endocr. J..

[4] Grundy Scott M., Cleeman James I., Daniels Stephen R., Donato Karen A., Eckel Robert H., Franklin Barry A., Gordon David J., Krauss Ronald M., Savage Peter J., Smith Sidney C. (2005). Diagnosis and Management of the Metabolic Syndrome. Circulation.

[5] Huang P.L. (2009). A comprehensive definition for metabolic syndrome. Dis. Model. Mech..

[6] Samson S.L., Garber A.J. (2014). Metabolic Syndrome. Endocrinol. Metab. Clin. N. Am..

[7] Malik M., Razig S.A. (2008). The prevalence of the metabolic syndrome among the multiethnic population of the United Arab Emirates: A report of a national survey. Metab. Syndr. Relat. Disord..

[8] Malik M., Bakir A., Saab B.A., Roglic G., King H. (2005). Glucose intolerance and associated factors in the multi-ethnic population of the United Arab Emirates: Results of a national survey. Diabetes Res. Clin. Pract..

[9] Ferguson J.F., Phillips C.M., Tierney A.C., Pérez-Martínez P., Defoort C., Helal O., Lairon D., Planells R., Shaw D.I., Lovegrove J.A. (2009). Gene-nutrient interactions in the metabolic syndrome: Single nucleotide polymorphisms in ADIPOQ and ADIPOR1 interact with plasma saturated fatty acids to modulate insulin resistance. Am. J. Clin. Nutr..

[10] Aljaibeji H.S., Mohammed A.K., Dhaiban S., Elemam N.M., Sulaiman N., Salehi A., Taneera J. (2019). Reduced expression of *PLCXD3* associates with disruption of glucose sensing and insulin signalling in pancreatic β-cells. Front. Endocrinol..

[11] Taneera J., Fadista J., Ahlqvist E., Atac D., Ottosson-Laakso E., Wollheim C.B., Groop L. (2014). Identification of novel genes for glucose metabolism based upon expression pattern in human islets and effect on insulin secretion and glycemia. Hum. Mol. Genet..

[12] Sulaiman N., Albadawi S., Abusnana S., Fikri M., Madani A., Mairghani M., Alawadi F., Zimmet P., Shaw J. (2015). Novel approach to systematic random sampling in population surveys: Lessons from the United Arab Emirates National Diabetes Study (UAEDIAB). J. Diabetes.

[13] Sulaiman N., Elbadawi S., Hussein A., Abusnana S., Madani A., Mairghani M., Alawadi F., Sulaiman A., Zimmet P., Huse O. (2017). Prevalence of overweight and obesity in United Arab Emirates Expatriates: The UAE National Diabetes and Lifestyle Study. Diabetol. Metab. Syndr..

[14] Sulaiman N., Mahmoud I., Hussein A., Elbadawi S., Abusnana S., Zimmet P., Shaw J. (2018). Diabetes risk score in the United Arab Emirates: A screening tool for the early detection of type 2 diabetes mellitus. BMJ Open Diabetes Res. Care.

[15] Dastani Z., Hivert M.F., Timpson N., Perry J.R., Yuan X., Scott R.A., Henneman P., Heid I.M., Kizer J.R., Lyytikainen L.P. (2012). Novel loci for adiponectin levels and their influence on type 2 diabetes and metabolic traits: A multi-ethnic meta-analysis of 45,891 individuals. PLoS Genet..

[16] Barrett J.C., Fry B., Maller J., Daly M.J. (2005). Haploview: Analysis and visualization of LD and haplotype maps. Bioinformatics.

[17] Kanai M., Akiyama M., Takahashi A., Matoba N., Momozawa Y., Ikeda M., Iwata N., Ikegawa S., Hirata M., Matsuda K. (2018). Genetic analysis of quantitative traits in the Japanese population links cell types to complex human diseases. Nat. Genet..

[18] Hebert H.L., Shepherd B., Milburn K., Veluchamy A., Meng W., Carr F., Donnelly L.A., Tavendale R., Leese G., Colhoun H.M. (2018). Cohort Profile: Genetics of Diabetes Audit and Research in Tayside Scotland (GoDARTS). Int. J. Epidemiol..

[19] Flannick J., Fuchsberger C., Mahajan A., Teslovich T.M., Agarwala V., Gaulton K.J., Caulkins L., Koesterer R., Ma C., Moutsianas L. (2017). Sequence data and association statistics from 12,940 type 2 diabetes cases and controls. Sci. Data.

[20] van der Heijden A.A., Rauh S.P., Dekker J.M., Beulens J.W., Elders P., M’t Hart L.M., Rutters F., van Leeuwen N., Nijpels G. (2017). The Hoorn Diabetes Care System (DCS) cohort. A prospective cohort of persons with type 2 diabetes treated in primary care in the Netherlands. BMJ Open.

[21] van Zuydam N.R., Ahlqvist E., Sandholm N., Deshmukh H., Rayner N.W., Abdalla M., Ladenvall C., Ziemek D., Fauman E., Robertson N.R. (2018). A Genome-Wide Association Study of Diabetic Kidney Disease in Subjects With Type 2 Diabetes. Diabetes.

[22] Wheeler E., Leong A., Liu C.T., Hivert M.F., Strawbridge R.J., Podmore C., Li M., Yao J., Sim X., Hong J. (2017). Impact of common genetic determinants of Hemoglobin A1c on type 2 diabetes risk and diagnosis in ancestrally diverse populations: A transethnic genome-wide meta-analysis. PLoS Med..

[23] Yengo L., Sidorenko J., Kemper K.E., Zheng Z., Wood A.R., Weedon M.N., Frayling T.M., Hirschhorn J., Yang J., Visscher P.M. (2018). Meta-analysis of genome-wide association studies for height and body mass index in approximately 700000 individuals of European ancestry. Hum. Mol. Genet..

[24] Mahajan A., Taliun D., Thurner M., Robertson N.R., Torres J.M., Rayner N.W., Payne A.J., Steinthorsdottir V., Scott R.A., Grarup N. (2018). Fine-mapping type 2 diabetes loci to single-variant resolution using high-density imputation and islet-specific epigenome maps. Nat. Genet..

[25] Bendl J., Musil M., Štourač J., Zendulka J., Damborský J., Brezovský J. (2016). PredictSNP2: A unified platform for accurately evaluating SNP effects by exploiting the different characteristics of variants in distinct genomic regions. PLoS Comput. Biol..

[26] Nsiah K., Shang V.O., Boateng K.A., Mensah F.O. (2015). Prevalence of metabolic syndrome in type 2 diabetes mellitus patients. Int. J. Appl. Basic Med. Res..

[27] Sperling L.S., Mechanick J.I., Neeland I.J., Herrick C.J., Despres J.P., Ndumele C.E., Vijayaraghavan K., Handelsman Y., Puckrein G.A., Araneta M.R. (2015). The CardioMetabolic Health Alliance: Working Toward a New Care Model for the Metabolic Syndrome. J. Am. Coll. Cardiol..

[28] Tune J.D., Goodwill A.G., Sassoon D.J., Mather K.J. (2017). Cardiovascular consequences of metabolic syndrome. Transl. Res..

[29] Verma P., Srivastava R.K., Jain D. (2018). Association of Lifestyle Risk Factors with Metabolic Syndrome Components: A Cross-sectional Study in Eastern India. Int. J. Prev. Med..

[30] Barroso I., McCarthy M.I. (2019). The Genetic Basis of Metabolic Disease. Cell.

[31] Fenwick P.H., Jeejeebhoy K., Dhaliwal R., Royall D., Brauer P., Tremblay A., Klein D., Mutch D.M. (2019). Lifestyle genomics and the metabolic syndrome: A review of genetic variants that influence response to diet and exercise interventions. Crit. Rev. Food Sci. Nutr..

[32] Gaulton K.J., Ferreira T., Lee Y., Raimondo A., Magi R., Reschen M.E., Mahajan A., Locke A., Rayner N.W., Robertson N. (2015). Genetic fine mapping and genomic annotation defines causal mechanisms at type 2 diabetes susceptibility loci. Nat. Genet..

[33] Wessel J., Chu A.Y., Willems S.M., Wang S., Yaghootkar H., Brody J.A., Dauriz M., Hivert M.F., Raghavan S., Lipovich L. (2015). Low-frequency and rare exome chip variants associate with fasting glucose and type 2 diabetes susceptibility. Nat. Commun..

[34] Chen P., Ong R.T., Tay W.T., Sim X., Ali M., Xu H., Suo C., Liu J., Chia K.S., Vithana E. (2013). A study assessing the association of glycated hemoglobin A1C (HbA1C) associated variants with HbA1C, chronic kidney disease and diabetic retinopathy in populations of Asian ancestry. PLoS ONE.

[35] Sim X., Ong R.T., Suo C., Tay W.T., Liu J., Ng D.P., Boehnke M., Chia K.S., Wong T.Y., Seielstad M. (2011). Transferability of type 2 diabetes implicated loci in multi-ethnic cohorts from Southeast Asia. PLoS Genet..

[36] Bishop M.T., Sanchez-Juan P., Knight R.S. (2013). Splice site SNPs of phospholipase *PLCXD3* are significantly associated with variant and sporadic Creutzfeldt-Jakob disease. BMC Med. Genet..

[37] Jamain S., Cichon S., Etain B., Mühleisen T.W., Georgi A., Zidane N., Chevallier L., Deshommes J., Nicolas A., Henrion A. (2014). Common and rare variant analysis in early-onset bipolar disorder vulnerability. PLoS ONE.

[38] Fischl A.M., Heron P.M., Stromberg A.J., McClintock T.S. (2014). Activity-dependent genes in mouse olfactory sensory neurons. Chem. Senses.

[39] Balendra R., Uphill J., Collinson C., Druyeh R., Adamson G., Hummerich H., Zerr I., Gambetti P., Collinge J., Mead S. (2016). Variants of *PLCXD3* are not associated with variant or sporadic Creutzfeldt-Jakob disease in a large international study. BMC Med. Genet..

[40] Barclay S.F., Rand C.M., Borch L.A., Nguyen L., Gray P.A., Gibson W.T., Wilson R.J., Gordon P.M., Aung Z., Berry-Kravis E.M. (2015). Rapid-Onset Obesity with Hypothalamic Dysfunction, Hypoventilation, and Autonomic Dysregulation (ROHHAD): Exome sequencing of trios, monozygotic twins and tumours. Orphanet J. Rare Dis..

[41] Suh P.-G., Park J.-I., Manzoli L., Cocco L., Peak J.C., Katan M., Fukami K., Kataoka T., Yun S., Ryu S.H. (2008). Multiple roles of phosphoinositide-specific phospholipase C isozymes. BMB Rep..

[42] Gellatly S.A., Kalujnaia S., Cramb G. (2012). Cloning, tissue distribution and sub-cellular localisation of phospholipase C X-domain containing protein (PLCXD) isoforms. Biochem. Biophys. Res. Commun..

